# Early-mid pregnancy renal parameters and adverse pregnancy outcomes in women with early stage CKD: a case series

**DOI:** 10.1007/s40620-025-02398-z

**Published:** 2025-08-26

**Authors:** Alessandra Orsillo, Erandi Hewawasam, Shilpanjali Jesudason

**Affiliations:** 1 Central and Northern Adelaide Renal and Transplantation Service, Adelaide, South Australia Australia; 2 Faculty of Health and Medical Sciences, The University of Adelaide, Adelaide, South Australia Australia; 3 Australia and New Zealand Dialysis and Transplant Registry, South Australian Health and Medical Research Institute, Adelaide, South Australia Australia

**Keywords:** Pregnancy, Renal insufficiency, Chronic, Pregnancy complications, Pregnancy outcome, Proteinuria, Hypertension, Pregnancy-induced

## Abstract

**Background:**

Early CKD may affect pregnancy outcomes, but identifying women at most risk remains challenging. We aimed to understand the predictive role of clinical parameters in early-mid pregnancy in women with early stage CKD.

**Methods:**

Women with CKD stage 1–3 with a pregnancy > 20 weeks gestation between 2018 and 2023 were evaluated for ‘red flag’ markers previously linked with risk of adverse pregnancy outcomes: failure of ≥ 10% fall in serum creatinine; urinary protein: creatinine ratio (uPCR) ≥ 30 mg/mmol in second trimester; lack of physiological fall in blood pressure by mid-pregnancy. The relationship between these red flags and a composite adverse pregnancy outcome of gestational age < 37 weeks, birth weight < 2500 g and pre-eclampsia was determined.

**Results:**

Of 38 mothers with 47 deliveries, 72% of pregnancies were in women with stage 1 CKD, 38% had hypertension and 19% had pre-eclampsia. Infants had median birth weight 2895 g (IQR: 2460–3170) and median gestational age 37.3 weeks (IQR 35.8–38). Serum creatinine did not fall ≥ 10% in 66% (*n* = 27/41) of women, uPCR was ≥ 30 mg/mmol in 69% (*n* = 24/35) and blood pressure did not fall in 73% (*n* = 24/33). Eighty-six percent had one or more ‘red flags’. The composite adverse pregnancy outcome occurred in 49% (*n* = 22/45). Women exhibiting any early-mid pregnancy red flags did not have increased rates of composite adverse pregnancy outcome (no creatinine fall, composite adverse pregnancy outcome *n* = 15, *p* = 0.176; proteinuria *n* = 15, composite adverse pregnancy outcome *p* = 0.066; no blood pressure fall, composite adverse pregnancy outcome *n* = 12, *p* = 1.00).

**Conclusions:**

The high rate of composite adverse pregnancy outcome in early stage CKD was not associated with traditional mid-pregnancy red flags. Best models of care for this cohort remain uncertain.

**Graphical abstract:**

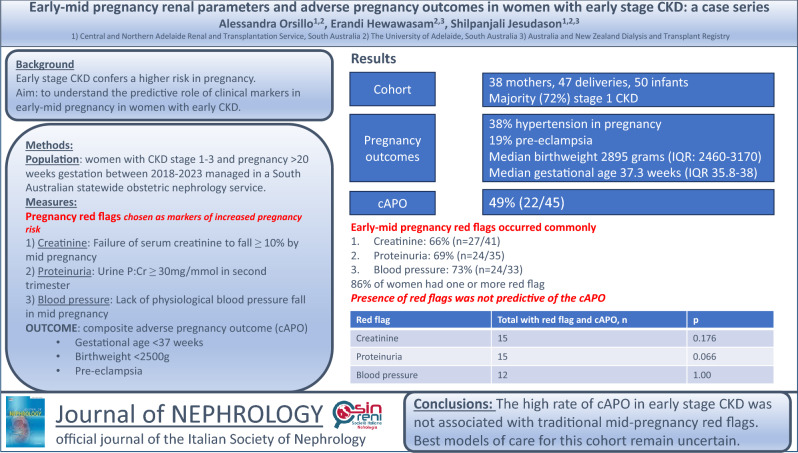

## Introduction

Chronic kidney disease (CKD) is an important public health problem, impacting 11% of adult Australians including women of reproductive age [1]. Early CKD (stages 1–2) may be under-recognised and under-diagnosed in pregnancy. Previous population-based studies have indicated that 7.5% of pregnancies occur in women with an estimated glomerular filtration rate (eGFR) between 60 and 90 mL/min [2].

The increased risk of adverse maternal and infant outcomes is well established in CKD. For CKD stages 3–5, these risks include preterm delivery and lower birth weight, as well as loss of kidney function in mothers [3]. Though later stages of CKD are recognised to contribute to higher pregnancy risk compared to early stage CKD, the significance of early stage CKD should not be underestimated [4–6]. Even in the absence of proteinuria and chronic hypertension—known factors which increase pregnancy risk—CKD stage 1 may confer a higher risk of adverse outcomes compared to the baseline population including lower gestational age at delivery and more admissions to neonatal intensive care units [6–8].

Challenges in the recognition of early stage CKD in pregnancy have contributed to a paucity of data on these women and their pregnancy outcomes. There is lack of clarity on the best models of care for this group that lead to early recognition of complications, while preventing over-medicalisation of lower risk pregnancies, which comes at a cost of high resource investment.

In the current study, we aimed to explore whether commonly accepted clinical markers in early to mid-pregnancy were informative regarding the risk of adverse pregnancy outcomes in women with early stage CKD.

## Methods

### Study design and setting

This was a retrospective, single-centre study of cases referred to the Obstetric Nephrology Service at the Royal Adelaide Hospital, Adelaide, South Australia. This service captures women referred for preconception counselling and antenatal care, and pregnancies are co-managed with high-risk pregnancy teams in maternity hospitals across South Australia. All women with CKD stages 1–3 who had a pregnancy between January 2018 and April 2023 were included.

### Participants

The inclusion criteria were women with CKD stages 1–3 of any cause. Preconception CKD was staged using CKD- EPI eGFR and proteinuria criteria [9]. If CKD was diagnosed in pregnancy, the stage of CKD in the first trimester was used. Only pregnancies > 20 weeks gestation were included, to enable the primary outcome. Women with CKD Stage 4/5 and kidney replacement therapy were excluded. Patients were followed for one year post pregnancy, where possible.

### Variables

Case data were collected as part of the South Australian pregnancy and CKD cohort study [10]. Data included demographic information, medical history and pregnancy history. Blood pressure, proteinuria (urine protein: creatinine ratio mg/mmol; [uPCR]) and creatinine were assessed pre-pregnancy and in the first, second and third trimester. Proteinuria and serum creatinine data were collected at 6 months and 12 months post-partum. Preconception hypertension was identified by searching case notes and prescription history. Outcomes sought were: the index pregnancy complications including pre-eclampsia and hypertension; delivery modality and complications; infant outcomes including pre-term birth and low birth weight. Data were missing for 5 pregnancies where patients sought care outside of the public hospital system and information was unavailable through electronic medical records. We identified and established ‘red flag’ markers known to be associated with worse pregnancy outcome [3, 6, 11]): (1) failure of serum creatinine to fall by 10% between pre-pregnancy and the second trimester; (2) pathological range uPCR ≥ 30 mg/mmol in the second trimester and (3) lack of physiological drop in blood pressure between pre-pregnancy and the second trimester. The primary outcome was the occurrence of a composite adverse pregnancy outcome of preterm birth with gestational age < 37 weeks, low birth weight < 2500 g and pre-eclampsia as defined by local guidelines [12].

### Statistical methods

Descriptive statistics were used to analyse the baseline characteristics and pregnancy outcomes, expressed as number of patients, or median and interquartile range. Categorical variables were expressed as percentages of the total cohort. Continuous variables were expressed as medians with an interquartile range.

Fisher’s exact test was used to assess the relationship between red flags and the composite adverse pregnancy outcome. Where missing data were present, these patients were excluded from the analysis.

The analyses were carried out using Stata/IC 18.0

### Ethics approval

Ethics approval was obtained from the Women’s and Children’s Health Network Human Research Ethics Committee in Adelaide, South Australia (2021/HRE00344).

## Results

### Included cases

We identified 38 mothers and 50 pregnancies with 47 live deliveries (44 singleton, 3 twin) and 3 miscarriages.

### Maternal demographics and kidney disease

Table 1 highlights the demographic data of mothers. The majority (*n* = 34, 72%) had stage 1 CKD at the time of pregnancy. The most common cause of CKD was glomerulonephritis: IgA glomerulonephritis (*n* = 9), minimal change disease (*n* = 3) and lupus nephritis (*n* = 3). Chronic hypertension was an existing comorbidity in 6 mothers. Twelve (24%) of the 50 pregnancies occurred via assisted reproductive technologies.

**Table 1:** Maternal demographics

Maternal characteristic		Result
Age at delivery, years, median (IQR)* ^a^		32.5 (30.4–35.3)
Stage of chronic kidney disease, *n*, (%)*	Stage 1	34 (72)
	Stage 2	8 (17)
	Stage 3	5 (11)
Cause of kidney disease, *n*^+^	Glomerulonephritis	23
	Reflux nephropathy	3
	Genetic disease	4
	Congenital cause	3
	Other	5
Pre-conception hypertension, *n*^+^		6
BMI at first pregnancy, kg/m^2^, median (IQR)^b^		28.0 (25.4–30)
Smoking status, *n*^+c^	Never	12
	Ex smoker	8
	Unknown	17
Previous pregnancies, *n*		28
Previous caesarean section, *n* (%)^d^		6 (16)

*Per pregnancy > 20 weeks (mothers may be counted twice)

^+^Per mother (captured at first pregnancy)

^a^Missing for *n* = 5

^b^Available for *n* = 14

^c^Missing for *n* = 1

^*d*^Missing for *n* = 1

### Obstetric, infant and maternal kidney outcomes

**Fig. 1:**
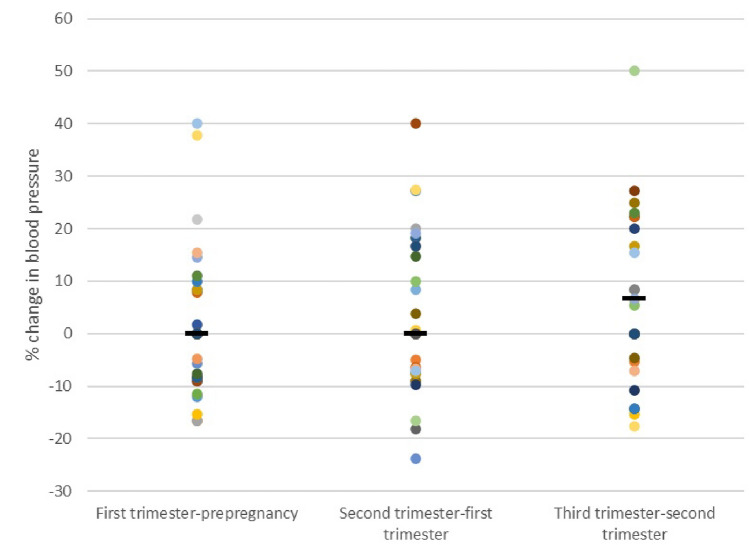
Percentage change in systolic blood pressure between time points across pregnancy in the early CKD cohort

Table 2 indicates complications during pregnancy with 18 (38%) mothers experiencing hypertension in pregnancy and 9 (19%) pre-eclampsia. Antihypertensive prescription is also detailed in this table. Of the total cohort, one patient was on two antihypertensives in the first trimester and two patients were on two antihypertensives in the second trimester. By the third trimester of pregnancy, five patients were on dual antihypertensives, with a further two patients requiring three antihypertensives. The percentage change in blood pressure in each trimester of pregnancy is shown in Fig. 1. Table 3 shows the median serum creatinine, uPCR, and blood pressure throughout each trimester of pregnancy, and follow up of proteinuria and serum creatinine at six and 12 months post-partum. The median increase in serum creatinine at 6 months post-partum was 2 µmol/L (IQR – 4–12) and at 12 months post-partum it was 0.5 µmol/L (IQR – 3.5–11). No women required dialysis in pregnancy or during the post-partum follow-up period.

**Table 2:** Pregnancy outcomes and adverse events

Pregnancy characteristic			Results
Composite adverse pregnancy outcome, *n*^a^			22
Gestational age, weeks, median (IQR)			37.3 (35.8–38)
Preterm birth total, *n* (%)			14 (30)
Gestational age, *n* (%)^b^	≥ 37 weeks		33 (69)
	32–37 weeks		13 (27)
	< 31 weeks		2 (4)
Birthweight, g, median (IQR)			2895 (2460–3170)
Pre-eclampsia, *n* (%)			9 (19)
Pregnancy hypertension, *n* (%)	Total		18 (38)
	Antihypertensives—first trimester	Methyldopa	1 (2)
		Labetalol	5 (11)
		Nifedipine	2 (4)
	Antihypertensives—second trimester	Methyldopa	1 (2)
		Labetalol	9 (19)
		Nifedipine	4 (9)
	Antihypertensives—third trimester	Methyldopa	6 (13)
		Labetalol	12 (26)
		Nifedipine	10 (21)
HELLP syndrome, *n* (%)			0 (0)
Eclampsia, *n* (%)			0 (0)
Gestational diabetes, *n* (%)			6 (13)
Placental abnormalities, *n* (%)			12 (26)
Small for gestational age/intrauterine growth restriction, *n* (%)			6 (13)
Antepartum haemorrhage, *n* (%)			1 (2)
Hospital admissions during pregnancy, *n* (%)			12 (26)
Mode of delivery, *n* (%)^c^	Vaginal		16 (40)
	Assisted		5 (13)
	C section		19 (48)
Type of C section, *n*	Planned		10
	Emergency		9
Special care after delivery, *n* (%)^d^			6 (16)

All data reported per pregnancy > 20 weeks/delivery aside from birthweight and special care, which is reported as per infant

^a^Missing for *n* = 2

^b^Missing for *n* = 2

^c^Missing for *n* = 7

^d^Missing for *n* = 13

**Table 3:** Renal parameters of clinical interest in pregnancy

Parameter		Results	Total pregnancies captured
Red flags of pregnancy, *n* (%)	No physiological drop of serum creatinine ≥ 10% pre-pregnancy—second trimester	27 (66)	41
	Second trimester PCR ≥ 30 mg/mmol	24 (69)	35
	No physiological fall in blood pressure between pre-pregnancy—second trimester	24 (73)	33
Number of red flags in pregnancy, *n*^a^	0	6	
	1	13	
	2	13	
	3	12	
cAPO incidence per number of red flags, *n* (%)	0	1 (17)	
	1^b^	5 (42)	
	2^c^	5 (42)	
	3	9 (75)	
Serum creatinine, µmol/L, median (IQR)	Pre pregnancy	66 (53–83)	43
	First trimester	60 (50–83)	40
	Second trimester	61 (54–85)	45
	Third trimester	66 (57–96)	44
	Delivery	74 (55–116)	30
	6 months post partum	72 (54–83)	38
	12 months post partum	64 (58–79)	27
Urinary P: Cr, mg/mmol, median (IQR)	Pre pregnancy	48 (18–104)	22
	First trimester	71 (37–120)	25
	Second trimester	41 (26–125)	35
	Third trimester	122 (49–331)	41
	Delivery	68 (46–223)	14
	6 months post partum	67 (24–115)	32
	12 months post partum	63 (20- 104)	21
Systolic blood pressure, mm/hg, median (IQR)	Pre pregnancy	120 (110–125)	33
	First trimester	113 (108–123)	36
	Second trimester	120 (100–140)	43
	Third trimester	125 (116–140)	37
Diastolic blood pressure, mm/hg, median (IQR)	Pre pregnancy	80 (65–80)	33
	First trimester	70 (60–80)	36
	Second trimester	80 (60–90)	43
	Third trimester	80 (65–90)	36

^a^Missing for *n* = 3

^b^Missing for *n* = 1

^c^Missing for *n* = 1

Of the cohort, six women continued immunosuppressive treatments during pregnancy. Diseases requiring immunosuppression included lupus nephritis, minimal change disease, focal segmental glomerulosclerosis, membranous nephropathy and haemolytic uraemic syndrome (HUS). Calcineurin inhibitors were the most commonly used oral agents (*n* = 4), followed by prednisolone (*n* = 2) and eculizumab for HUS management (*n* = 1). Two patients received plasma exchange throughout pregnancy, one was for management of primary kidney disease and the other for treatment of a concurrent haematological disorder. In this group on immunosuppressive therapy, 5 had the composite adverse pregnancy outcome.

There was a total of 14 (30%) preterm births, a median gestational age of 37.3 weeks and median birth weight of 2895 g (Table 2). For the 20 newborns whose Apgar scores were known, 14 (70%) had an Apgar score of 9 at 5 minutes, and 17 (85%) had an APGAR of 9 at 10 minutes post-delivery.

### Early-mid pregnancy ‘red flag’ markers and the composite adverse outcome

The composite adverse pregnancy outcome, defined as birth weight < 2500 g, gestational age < 37 weeks or pre-eclampsia, occurred in 22 of 45 pregnancies (49%). Among women with the composite adverse pregnancy outcome, the median age was 34 years (IQR 31–38 years), and 19 of these women had one or more red flags.

In the overall group, serum creatinine failed to decrease by at least 10% between pre-pregnancy and second trimester in 27/41 pregnancies (66%). uPCR ≥ 30 mg/mmol in the second trimester was observed in 24/35 (69%) women. The median second trimester uPCR for the group was 41 mg/mmol (IQR 26–125). Systolic blood pressure did not fall physiologically in the second trimester for 24/33 (73%) pregnancies, while it increased ≥ 10% in 10/33 (30%) pregnancies. Overall, at least one pre-assigned predictor of pregnancy risk was present in 38/44, or 86% of all pregnancies where data were captured.

Among women with one red flag, 5/12 (42%) had composite adverse pregnancy outcome. For those with two red flags, 5/12 (42%) had composite adverse pregnancy outcome, and among women with all three red flags, 9/12 (75%) had composite adverse pregnancy outcome. For the 6 pregnancies without any red flag markers, only one had composite adverse pregnancy outcome.

When investigating the relationship between any particular red flag and composite adverse pregnancy outcome, no statistically significant relationship was found. Among those whose serum creatinine failed to decline by 10%, composite adverse pregnancy outcome occurred in 15 subjects, however, this red flag did not indicate a higher likelihood of composite adverse pregnancy outcome compared to those with a normal physiological creatinine decline (*p* = 0.176). Similarly, composite adverse pregnancy outcome was not more frequent among women with a pathological uPCR ≥ 30 mg/mmol (*n* = 15, *p* = 0.066) or those who lacked the expected second trimester blood pressure drop (*n* = 12, *p* = 1.0).

## Discussion

This investigation harnessed detailed data from pregnancies in women with early stage CKD to understand the predictive abilities of renal parameters readily accessible in a clinical setting in informing overall pregnancy risk. Though these women were predominantly stage 1 CKD and had few other medical comorbidities, pregnancy complications in this group remained high. Data from this group of patients are consistent with our understanding that these women remain at above average risk during pregnancy, with high rates of gestational hypertension, pre-eclampsia and early delivery. Furthermore, this study focussed on a pragmatic exploration of the prevalence of well established ‘red flags’—common clinical measures in women with early CKD.

Despite the high burden of renal adverse markers amongst the group of patients (1 or more marker observed in 38/44, or 86% of pregnancies), women with these red flags did not have statistically significant increased occurrence of the composite adverse pregnancy outcome. These data add to current knowledge about risk stratification in women with early stage CKD.

Our cohort of patients had higher rates of complications in pregnancy. Pregnancy hypertension occurred in 38% of pregnancies. In contrast, 0.9% of Australian mothers had antenatal hypertension, and gestational hypertension occurred in 3.1% of Australian pregnancies in 2022 [13]. Pre-eclampsia occurred in 19%, compared to the national average of 3.3% [14]. In our group, 31% of deliveries were before 37 weeks, which is much higher than in the general Australian population (8.7%) [13]. Similar small studies have sought to characterise this group of early stage CKD patients and their risk in pregnancy. An Australian study of 55 women across all stages of CKD included 27 women with stage 1–2 CKD; 41% with chronic hypertension and 59% with proteinuria. In this study, adverse outcomes were common, with preeclampsia in 63% compared to the control population of 4.1%. Fifty-six percent of infants were delivered pre-term, and the same percentage was delivered by caesarean section. Fourteen infants (52%) required neonatal intensive care after delivery [15]. However, this may reflect selection of high-risk patients, cared for in specialist services, in small cohort studies. On a larger population scale there may be many women with early stage CKD who are at population risk. In a large study by Tangren et al., including 42,543 women with stage 1–2 CKD and 479 with eGFR between 45 and 60 mL/min, there was no difference in a series of maternal morbidity outcomes between those with stage 1–2 CKD compared to the general population. Adverse infant outcomes, including preterm delivery rates, increased with worsening CKD stage [2].

In our study, there was little change in serum creatinine post-partum. There are few studies that explore the change in serum creatinine post-partum in early stage CKD. A systematic review by Zhang et al. concluded that the group of early stage CKD patients may not experience a decline in kidney function following pregnancy [16]. A smaller study by He et al. suggested that eGFR does not decline in women with CKD 3a if proteinuria is < 1 g/24 h [17]. Our results are largely consistent with this, where patient follow up data were available, which is reassuring to women with early stage CKD considering pregnancy.

The risk of proteinuria in pregnancies has been well documented in large population-based studies. The risk attached to proteinuria escalates with the severity of proteinuria [18]. In early pregnancy, nephrotic range proteinuria is linked to pre-term birth and poorer long term maternal kidney function [19]. However, even in stage 1 chronic kidney disease, proteinuria > 1 g/24 h is associated with low birth weight and early pre term births [2, 8]. In our cohort, mid-pregnancy proteinuria did not show a significant association with the composite adverse pregnancy outcome, however this may reflect lower grade proteinuria or smaller sample size.

This study captured blood pressure data prior to and throughout pregnancy. A recent meta-analysis examining physiological blood pressure changes in pregnancy observed mean systolic blood pressure nadir at 10 weeks gestation, and mean diastolic blood pressure nadir at 21 weeks gestation [20], while another study indicated a systolic blood pressure nadir at 18.6 weeks, and diastolic nadir at 19.2 weeks [11]. We hypothesised that CKD patients without this normal physiological adaptation may be at higher risk during pregnancy. In our cohort, a large proportion (24/33 patients) did not exhibit a physiological drop in blood pressure. This failure of blood pressure to fall, or a rise in blood pressure, was not associated with higher rates of the composite adverse pregnancy outcome, but overall this group had physiological median systolic and diastolic pressures (< 140/90) [12]. This likely reflects their management in a specialised service with close monitoring. Chronic hypertension is known to increase rates of pre-term delivery, with this risk compounded if serum creatinine does not fall in pregnancy [3]. Our cohort included a relatively small proportion of patients with chronic hypertension (*n* = 6), limiting our ability to make conclusions around this group.

Our study has sought to examine clinical parameters as risk markers that can aid the individual clinician in guiding women through their pregnancy risks. Given that clinical markers were not predictive of risk in our group, we expect that these decisions may be aided by biochemical measures, including measurement of placental angiogenic biomarkers used to predict pre-eclampsia [21]. Our understanding of the use of these markers in CKD continues to evolve [22].

A strength of this study is the granular data on blood pressure, proteinuria and creatinine changes throughout pregnancy. These data are usually missing from administrative or perinatal datasets that are not focussed on CKD. The limitations include those inherent to smaller single-centre studies, retrospective data collection, and the potential selection bias related to referral to an obstetric nephrology expert centre. Our study did not capture fetal growth data, placental ultrasound or angiogenic parameters, nor was it powered for more complex subgroup analyses. This study does represent the first exploratory analysis of a new, state-wide cohort study focussed on pregnancy and CKD [10] with plans for national collaborations and data pooling. In time, sufficient case numbers will be acquired to overcome these limitations. Finally, it is possible that having women with earlier CKD in a specialised obstetric nephrology service could lead to over-testing and over-medicalisation of pregnancy, with clinician nervousness about prolonging pregnancy, thereby driving iatrogenic preterm birth. This is only postulated, but underscores the imperative to develop the evidence base for the most appropriate care of this cohort of women. The current study focussed only on mid-pregnancy changes as this is where pre-emptive action may be of benefit, and future work will explore the events closer to delivery to understand the clinical trajectory further.

This research outlines the value of detailed data sets in obstetric patients with early CKD. By understanding the predictors of pregnancy, infant and maternal risk will offer valuable insights to guide models of care and referral processes. While this knowledge is being developed, at present, models of care are likely to be driven by access to local expertise and guidelines, and the care of patients should ideally be undertaken within a multidisciplinary team. Within our service, we use local expertise to offer preconception counselling and risk assessment to individual patients, which drives a ‘shared care’ approach between our obstetric nephrology service, obstetricians and patients’ primary nephrologists. The frequency of visits is dictated on a case-by-case basis.

Ultimately, resources may not allow triage of all early stage CKD patients to high risk pregnancy clinics, therefore it is critical to establish early which patients need additional care throughout pregnancy. Our study highlights the value of prospective, detailed data capture of clinical parameters that on a larger scale, might aid in this clinical decision making. Our ongoing state-wide cohort study will assist in providing these insights and inform us around pregnancy outcomes in a contemporary CKD cohort.

In conclusion, this study reports a group of patients with early stage CKD who demonstrated high risk of adverse pregnancy outcome, where traditional ‘red flag markers’ in early to mid-pregnancy were prevalent but not always predictive of adverse outcomes. Women with early stage CKD should be counselled with regard to the increased risk of pregnancy complications and the need for vigilance during pregnancy.

## Data Availability

The datasets generated during and/or analysed during the current study are available from the corresponding author on reasonable request.
